# Age, rather than hypertension duration, drives coronary endothelial degradation: an *in situ *post-mortem analysis

**DOI:** 10.1186/s12872-026-06210-z

**Published:** 2026-07-01

**Authors:** Oleh Samchuk, Yevhen Panasyuk, Vladyslav Bardash, Orest Zolotukhin, Eugen Sklyarov, Igor Skrypnyk

**Affiliations:** 1 St. Panteleimon Hospital, First Lviv Medical Union, Lviv, Ukraine; 2 Department of Therapy No. 1, Medical Diagnostics, Hematology, and Transfusiology, Faculty of Postgraduate Education, Danylo Halytsky Lviv National Medical University, Lviv, Ukraine; 3 Department of Internal Medicine No. 1, Poltava State Medical University, Poltava, Ukraine

**Keywords:** Vascular aging, Essential hypertension, Endothelial glycocalyx, Syndecan-1, PECAM-1, Digital pathology

## Abstract

**Background:**

Arterial hypertension drives coronary vascular remodeling, yet disentangling the independent effects of physiological aging and chronic hemodynamic overload on the endothelium (CD31) and glycocalyx (CD138) in situ remains challenging. Most clinical studies evaluate soluble circulating markers, while direct morphological evidence of tissue-level spatial degradation is scarce.

**Methods:**

This observational post-mortem study evaluated coronary artery fragments from 30 deceased patients (10 controls, 20 with essential hypertension) using immunohistochemistry and digital pathology. To mitigate confounding bias caused by age discrepancies and acute pre-mortem systemic stressors in the control group (e.g., fatal trauma), multivariable linear regression modeling with robust standard errors was applied exclusively to the hypertensive cohort to isolate the independent impacts of chronological age and hypertension duration.

**Results:**

Within the hypertensive cohort, chronological age emerged as a significant independent factor inversely associated with CD31 expression area (β = -0.74, 95% CI: -0.98 to -0.50, *p* = 0.016). The duration of hypertension was not independently associated with CD31 loss (*p* = 0.076). Furthermore, the multivariable model for CD138 did not reach overall statistical significance (for age: β = -0.17, 95% CI: -0.42 to 0.07, *p* = 0.200; for hypertension duration: β = 0.21, 95% CI: -0.06 to 0.48, *p* = 0.130). However, a robust positive correlation was observed between CD31 and CD138 tissue expression levels (*R* = 0.50, *p* = 0.025), indicating synchronized structural degradation.

**Conclusions:**

Chronological age, rather than the chronicity of hypertension, is significantly associated with reduced CD31 expression in the coronary arteries of hypertensive patients. The positive correlation between CD31 and CD138 expression highlights a synchronized spatial degradation of the endothelium and its protective glycocalyx. These findings highlight the critical necessity of isolating physiological senescence from pathological remodeling in vascular research.

**Supplementary Information:**

The online version contains supplementary material available at https://doi.org/10.1186/s12872-026-06210-z.

## Background

Arterial hypertension remains the leading risk factor for cardiovascular disease and related mortality worldwide [1, 2]. A key pathogenetic link in hypertensive complications is the structural remodeling of the vasculature, particularly the coronary arteries, which eventually leads to ischemic heart disease and microvascular dysfunction [3]. The primary target for hemodynamic stress is the endothelium [4], whose functional and structural alterations represent the initial stage of vascular injury, preceding the clinical manifestations of atherosclerosis and myocardial hypertrophy [5].

A critical structure ensuring the homeostasis and mechanotransduction of endothelial cells is the endothelial glycocalyx — a complex layer of macromolecules on the luminal surface of vessels [6, 7]. Syndecan-1 (CD138) is the primary transmembrane proteoglycan of the glycocalyx, responsible for its stability and known to degrade rapidly under chronic hemodynamic load [8]. In turn, platelet endothelial cell adhesion molecule-1 (CD31) serves as a reliable histological marker of endothelial monolayer integrity [9], with changes in its expression closely correlating with vascular remodeling and chronological aging of the arterial wall [10].

Despite the profound understanding of these processes, the existing evidence base has significant limitations [11, 12]. Most clinical studies focus on evaluating soluble syndecan-1 in the blood as a systemic biomarker of glycocalyx degradation, while direct immunohistochemical studies of the spatial distribution of CD31 and CD138 directly in human coronary artery tissues in situ remain scarce [13]. Furthermore, morphological cohort studies encounter the complex issue of confounding bias between physiological vascular aging and the pathological impact of hypertension duration [14, 15], which continues to hinder the determination of their independent contributions to the degradation of the endothelial barrier.

Given these gaps in knowledge, the primary objective of this study was to evaluate the spatial expression patterns of CD31 and CD138 in the coronary arteries of patients with essential hypertension compared to a non-hypertensive control group. Furthermore, we aimed to apply multivariable regression modeling within the hypertensive cohort to disentangle the independent effects of chronological age and hypertension duration on endothelial dysfunction and glycocalyx degradation at the tissue level.

## Materials and methods

This study was designed as an observational comparative post-mortem analysis. The study material consisted of coronary artery fragments harvested during autopsies. Specifically, the analysis was restricted exclusively to the branches of the left coronary system (left anterior descending) to minimize confounding bias arising from differing regional hemodynamic profiles. The research utilized archived biological material obtained during standard pathological or forensic autopsy procedures, without any interventions on the deceased beyond the standard protocol. All personal data were strictly anonymized prior to analysis. The study was conducted in accordance with the principles of the Declaration of Helsinki and current Ukrainian legislation regarding bioethics and human tissue research.

The inclusion criteria for the study were as follows: (1) age of the deceased ≥ 18 years; (2) sufficient volume and structural preservation of coronary arteries for histological and immunohistochemical analysis; (3) documented clinical history of essential hypertension (for the primary study group) or its confirmed absence (for the control group); and (4) a post-mortem interval not exceeding 48 h.

The diagnosis, staging, and grading of essential hypertension were established in accordance with the 2023 ESH Guidelines for the management of arterial hypertension.

Specimens were excluded based on the following criteria: (1) presence of systemic vasculitis or diffuse connective tissue diseases; (2) clinical evidence of sepsis or systemic inflammatory response syndrome (SIRS) prior to death; (3) oncological diseases with direct cardiac or vascular involvement; and (4) pronounced signs of vascular wall autolysis.

Specifically for the control group, to ensure that the observed vascular morphology was not confounded by other pathologies, the cohort exclusively comprised previously healthy individuals who died suddenly from acute trauma. The strict absence of undiagnosed cardiovascular, genetic, metabolic, or chronic inflammatory diseases was confirmed through a comprehensive review of their pre-mortem outpatient medical records and history of clinical visits. For the control group, the confirmed absence of any concomitant cardiovascular pathologies — including ischemic heart disease, cardiomyopathies, heart failure, and valvular defects — was a strict requirement. Additionally, according to the clinical and autopsy records, the primary cause of death for all patients in the main hypertensive cohort was acute fatal stroke.

The sample size was not determined via a priori statistical calculation but relied on a convenience sampling strategy. This approach was dictated by the severe inherent limitations in acquiring well-preserved human post-mortem coronary tissue within a 48-hour post-mortem interval, strictly matching our rigorous inclusion and exclusion criteria (especially the complete absence of incidental cardiometabolic diseases in the control group). To estimate the robustness of our findings, a post hoc power analysis was performed for the primary multivariable linear regression models utilizing the pwr package in R. For the hypertensive cohort (*n* = 20) with two tested predictors (age and disease duration), the observed statistical power (1-β) based on Cohen’s $\:{f}^{2}$ effect sizes was 66.2% for the CD31 model and 31.1% for the CD138 model, assuming an $\:\alpha\:$ level of 0.05.

Demographic and clinical data, including sex, age, cause of death, and comorbidities, were systematically extracted from terminal inpatient medical records and autopsy reports. Laboratory parameters reflect blood samples collected upon emergency hospital admission, typically within 24 to 48 h prior to death, representing the acute terminal clinical status of the patients.

Coronary artery fragments were fixed in 10% neutral buffered formalin for 24 to 48 h. Following standard automated tissue processing, the samples were embedded in paraffin blocks. For each evaluated coronary artery, three consecutive (serial) four-micrometer-thick histological sections were prepared to allow spatial alignment of morphological features. These serial sections were subjected to routine hematoxylin and eosin (H&E) staining and immunohistochemistry (IHC) for CD31 and CD138, respectively.

For the IHC analysis, the following primary antibodies were utilized: GeneAb Monoclonal Mouse Anti-Human CD31 Antibody (ref. IHC031-7) and GeneAb Monoclonal Mouse Anti-Human CD138 Antibody (ref. IHC138-7). To ensure maximum technical consistency and eliminate batch-to-batch variability for subsequent digital quantification, all immunohistochemical stainings were performed simultaneously in a single batch under strictly standardized conditions. Briefly, tissue sections (4 μm) were incubated at 60 °C for 1 h, deparaffinized in two changes of xylene, and rehydrated through graded alcohol solutions. Heat-induced epitope retrieval was performed using EDTA buffer (pH 9.0) preheated to 98 °C for 30–40 min. Endogenous peroxidase activity was blocked with 3% hydrogen peroxide for 10 min, followed by the application of a protein blocking reagent for 5–10 min. Sections were then incubated with the respective primary antibodies for 30 min at room temperature. Signal amplification was achieved using a post-block reagent (20 min) and an HRP-polymer detection system (30 min). Immunoreactivity was visualized using 3,3′-diaminobenzidine (DAB) chromogen (prepared at a 1:20 dilution) for 10 min. Finally, sections were counterstained with hematoxylin for 5 min, dehydrated, cleared in xylene, and mounted. The specificity of the staining was rigorously validated utilizing internal negative controls; non-target structures (e.g., vascular smooth muscle cells in the tunica media and adipocytes) consistently demonstrated a complete absence of nonspecific DAB deposition across all analyzed sections.

The histological slides were digitized using a Morpholens 1 whole-slide scanner. The resulting whole-slide images were evaluated using QuPath open-source software, version 0.6.0. To minimize observer bias, the digital image annotation and quantitative analysis were performed by an investigator (O.Z.) who was completely blinded to the clinical group allocations. The quantity of the chromogen signal in the coronary arteries was measured using a thresholding function and expressed as the area of positive staining relative to the total annotated area of the endothelium and subendothelial matrix. Subsequent data aggregation and statistical modeling were conducted independently by a separate investigator (V.B.). To differentiate specific signals from non-specific background noise, a predefined staining intensity threshold of 0.35 was applied. This value was determined empirically during preliminary visual validation to provide the optimal balance between sensitivity and specificity, explicitly preventing the false-positive inclusion of non-specific background and hematoxylin overlap resulting from the broad spectral distribution of 3,3’-diaminobenzidine (DAB) chromogen across red, green, and blue (RGB) color channels (Supplementary Fig. S1). To ensure methodological reproducibility, this single fixed threshold was locked and applied uniformly across all digitized whole-slide images. The endothelial and subendothelial matrix regions were manually annotated using the “brush” tool at high magnification (50×).

Additionally, a semi-quantitative assessment of arterioles within the adjacent adipose tissue was performed. A scoring system was applied based on the proportion of arterioles exhibiting circumferential chromogen accumulation that covered more than 75% of the endothelial circumference: 0 points (0% of arterioles); 1 point (1–24%); 2 points (25–49%); 3 points (50–74%); and 4 points (75–100%).

Data aggregation and initial systematization were performed using Microsoft Excel 2016. Advanced statistical modeling and visualization were conducted using the R programming environment (version 4.5.3). Missing data points within the routine biochemical panel were addressed using Multiple Imputation by Chained Equations. Notably, this imputation protocol was restricted exclusively to laboratory parameters and was not applied to the primary immunohistochemical targets or morphological markers.

Due to the limited sample size and non-normal distribution of the data, continuous variables were summarized as the median and interquartile range (IQR). Categorical variables were presented as absolute counts and percentages, supplemented with 95% confidence intervals (CI) calculated using the Wilson score interval method. Intergroup comparisons were performed using the non-parametric Mann-Whitney U test for continuous variables and Fisher’s exact test for categorical variables.

Bivariate relationships between continuous and ordinal variables were evaluated using Spearman’s rank-order correlation coefficient, with trends visually represented via Locally Estimated Scatterplot Smoothing curves. To identify independent factors associated with CD31 and CD138 expression areas, multiple linear regression models were constructed exclusively within the hypertensive cohort to prevent confounding bias.

Comprehensive regression diagnostics were performed to ensure model validity. The absence of multicollinearity among predictors was confirmed using the Variance Inflation Factor (VIF < 2.0). The assumptions of residual normality and homoscedasticity were verified using the Shapiro-Wilk and Breusch-Pagan tests, respectively. Residual autocorrelation was assessed via the Durbin-Watson test. For models demonstrating significant autocorrelation (*p* < 0.05), Newey-West Heteroskedasticity and Autocorrelation Consistent robust standard errors were applied to compute adjusted 95% CIs and *p* values, ensuring reliable statistical inference. All statistical tests were two-sided, and a *p* value of < 0.05 was considered statistically significant.

## Results

A total of 30 patients were enrolled in the study and stratified into a control group (without arterial hypertension, *n* = 10) and a primary study group (with essential hypertension, *n* = 20) (Table 1). The cohorts were well-matched regarding sex distribution (*p* = 0.682), with males comprising 80% and 70% of the control and hypertension groups, respectively.

**Table 1:** Baseline demographic, clinical, and laboratory characteristics of the study patients

Characteristic	Control (*n* = 10)	Hypertension (*n* = 20)	*P* value
Demographics			
Age (years)	37.00 (31.00–38.00)	72.50 (67.00–76.00)	**< 0.001**
Sex			0.682
— Female	2 (20.0%) [95% CI: 3.5–55.8]	6 (30.0%) [95% CI: 12.8–54.3]	
— Male	8 (80.0%) [95% CI: 44.2–96.5]	14 (70.0%) [95% CI: 45.7–87.2]	
Clinical parameters			
Hypertension stage 3	0 (0%)	20 (100%) [95% CI: 80.0–100]	**< 0.001**
Hypertension grade			**< 0.001**
— Grade 1 *(140–159 / 90–99 mmHg)*	0 (0%)	2 (10.0%) [95% CI: 1.8–33.1]	
— Grade 2 *(160–179 / 100–109 mmHg)*	0 (0%)	4 (20.0%) [95% CI: 6.6–44.3]	
— Grade 3 *(≥ 180 / ≥110 mmHg)*	0 (0%)	14 (70.0%) [95% CI: 45.7–87.2]	
HTN duration (years)	NA	15.00 (12.00–19.00)	—
Morphological markers			
CD31 Expression area in arteries (%)	8.01 (5.83–16.00)	8.95 (3.77–12.33)	0.912
CD31 expression in arterioles			0.200
— 0%	0 (0%) [95% CI: 0.0–34.0]	0 (0%) [95% CI: 0.0–20.0]	
— <25%	0 (0%) [95% CI: 0.0–34.0]	2 (10.0%) [95% CI: 1.8–33.0]	
— 26–50%	0 (0%) [95% CI: 0.0–34.0]	2 (10.0%) [95% CI: 1.8–33.0]	
— 51–75%	0 (0%) [95% CI: 0.0–34.0]	4 (20.0%) [95% CI: 6.6–44.0]	
— 76–100%	10 (100%) [95% CI: 66.0–100]	12 (60.0%) [95% CI: 36.0–80.0]	
CD138 Expression area in arteries (%)	2.00 (0.00–3.06)	2.28 (0.52–5.15)	0.841
CD138 expression in arterioles			> 0.999
— 0%	2 (20.0%) [95% CI: 3.5–56.0]	5 (25.0%) [95% CI: 9.6–49.0]	
— <25%	2 (20.0%) [95% CI: 3.5–56.0]	5 (25.0%) [95% CI: 9.6–49.0]	
— 26–50%	2 (20.0%) [95% CI: 3.5–56.0]	2 (10.0%) [95% CI: 1.8–33.0]	
— 51–75%	2 (20.0%) [95% CI: 3.5–56.0]	5 (25.0%) [95% CI: 9.6–49.0]	
— 76–100%	2 (20.0%) [95% CI: 3.5–56.0]	3 (15.0%) [95% CI: 4.0–39.0]	
Laboratory findings			
Hemoglobin (g/L)	85.00 (80.00–130.00)	143.50 (129.50–156.50)	**0.004**
Red blood cells (×10¹²/L)	2.85 (2.69–4.12)	4.75 (4.38–5.08)	**< 0.001**
White blood cells (×10⁹/L)	14.57 (9.72–18.72)	13.96 (12.59–16.90)	0.800
Platelets (×10⁹/L)	198.00 (72.00–288.00)	290.00 (197.50–341.00)	0.078
AST (U/L)	171.00 (83.30–702.00)	47.30 (24.75–96.90)	**0.013**
ALT (U/L)	152.00 (32.80–1932.00)	21.80 (18.55–25.70)	**0.009**
Total bilirubin (µmol/L)	13.90 (7.10–66.40)	20.95 (6.90–47.60)	> 0.999
Total protein (g/L)	67.85 (47.80–69.90)	69.45 (67.85–71.60)	0.120
Creatinine (µmol/L)	116.00 (61.60–213.00)	123.00 (89.90–294.00)	0.400
Urea (mmol/L)	10.05 (5.32–49.50)	10.28 (6.33–77.90)	0.600
C-reactive protein (mg/L)	11.90 (9.03–75.10)	44.05 (9.03–124.10)	0.600
Fibrinogen (g/L)	4.44 (2.88–4.44)	3.44 (2.88–4.32)	0.400

Continuous variables are presented as median (interquartile range [IQR]). Categorical variables are presented as absolute counts (percentages) with 95% confidence intervals (CI) calculated using the Wilson score interval

*HTN* Hypertension, *AST* Aspartate Aminotransferase, *ALT* Alanine Aminotransferase. Non-significant coagulation profile data were omitted for brevity but are available upon request

*P*-values were calculated using the Mann-Whitney U test for continuous variables and Fisher’s exact test for categorical variables. Significant P values (< 0.05) are highlighted in bold

The primary distinguishing baseline characteristics between the cohorts were age and cardiovascular status. Patients in the hypertension group were significantly older (median 72.5 [IQR: 67.0–76.0] vs. 37.0 [31.0–38.0] years, *p* < 0.001). Within the primary study group, 100% of patients presented with stage 3 hypertension, and 70% exhibited grade 3 elevated blood pressure. The median duration of the hypertensive history in this cohort was 15.0 (12.0–19.0) years.

**Fig. 1:**
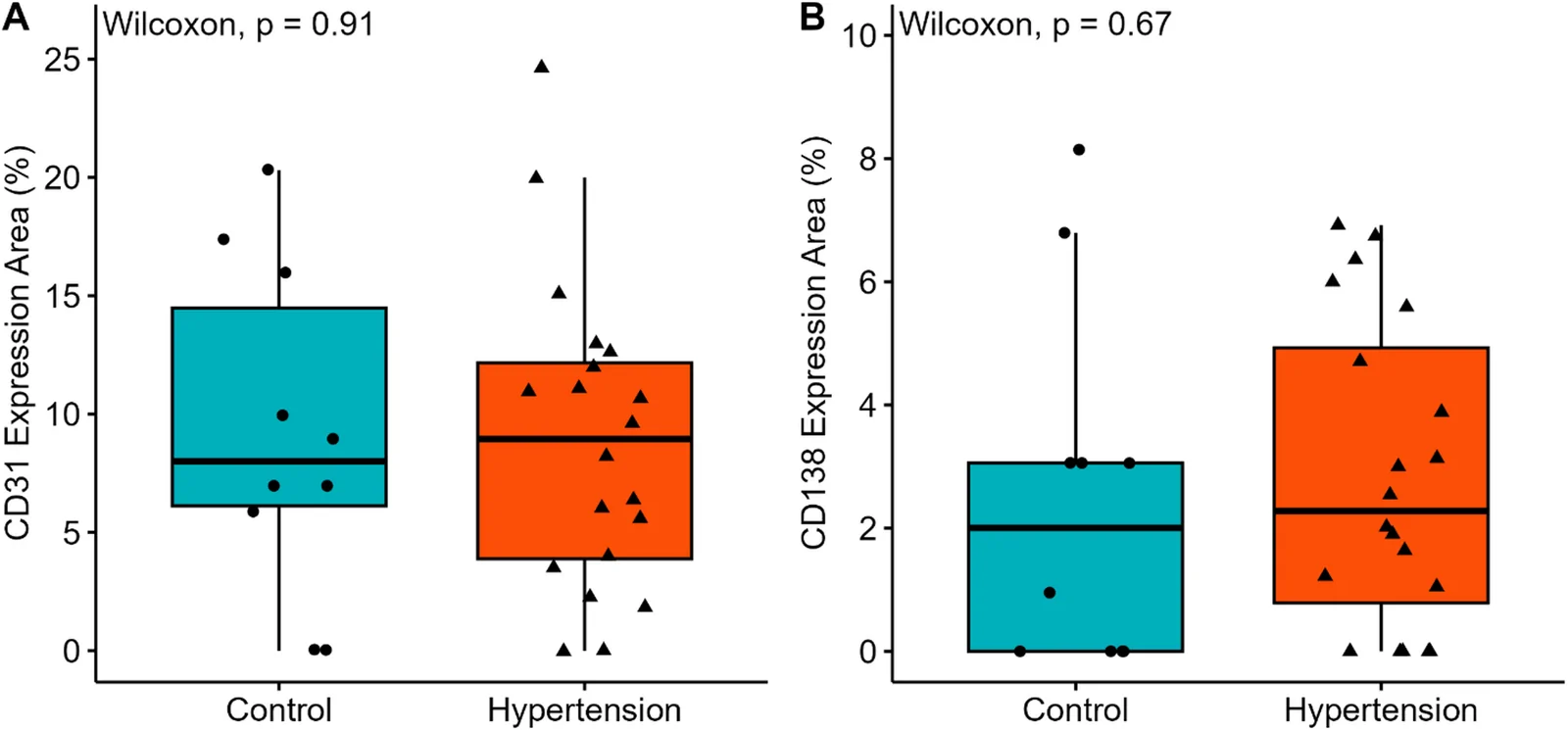
Expression of morphological markers in arterial vessels. **A** CD31 expression area (%) in the Control and Hypertension groups. **B** CD138 expression area (%) in the respective groups. Box plots display the median (thick horizontal line) and interquartile range (IQR, hinges). Whiskers extend to the most extreme data points no further than 1.5 × IQR from the hinge. Individual patient data points are overlaid to demonstrate data distribution (control, *n* = 10; hypertension, *n* = 20). Statistical comparisons were performed using the two-sided Wilcoxon rank-sum test

Direct comparative analysis of the primary immunohistochemical targets did not reveal statistically significant intergroup differences at baseline (Fig. 1). The expression area of the endothelial marker CD31 in arteries was 8.95% (3.77–12.33%) in the hypertension group versus 8.01% (5.83–16.00%) in the control cohort (*p* = 0.912). Similarly, syndecan-1 (CD138) expression areas remained comparable between the groups (2.28% [0.52–5.15%] vs. 2.00% [0.00–3.06%], *p* = 0.841). The categorical distribution of marker expression in small arterioles also demonstrated no significant variance between the cohorts for both CD31 (*p* = 0.200) and CD138 (*p* >0.999) (Table 1), underscoring the necessity of subsequent regression modeling to account for age-related confounding.

While the majority of routine biochemical and coagulation panels (including total protein, creatinine, urea, and fibrinogen) were comparable between the groups, specific significant differences were identified. The control group exhibited significantly elevated hepatic transaminases, with median AST at 171.0 (83.3–702.0) U/L compared to 47.3 (24.75–96.9) U/L in the hypertension group (*p* = 0.013), and ALT at 152.0 (32.8–1932.0) U/L versus 21.8 (18.55–25.70) U/L (*p* = 0.009). Additionally, the hypertension cohort demonstrated significantly higher red blood cell (RBC) counts (4.75 [4.38–5.08] vs. 2.85 [2.69–4.12] ×10¹²/L, *p* < 0.001) and hemoglobin levels (143.5 [129.5–156.5] vs. 85.0 [80.0–130.0] g/L, *p* = 0.004) compared to controls.

**Fig. 2:**
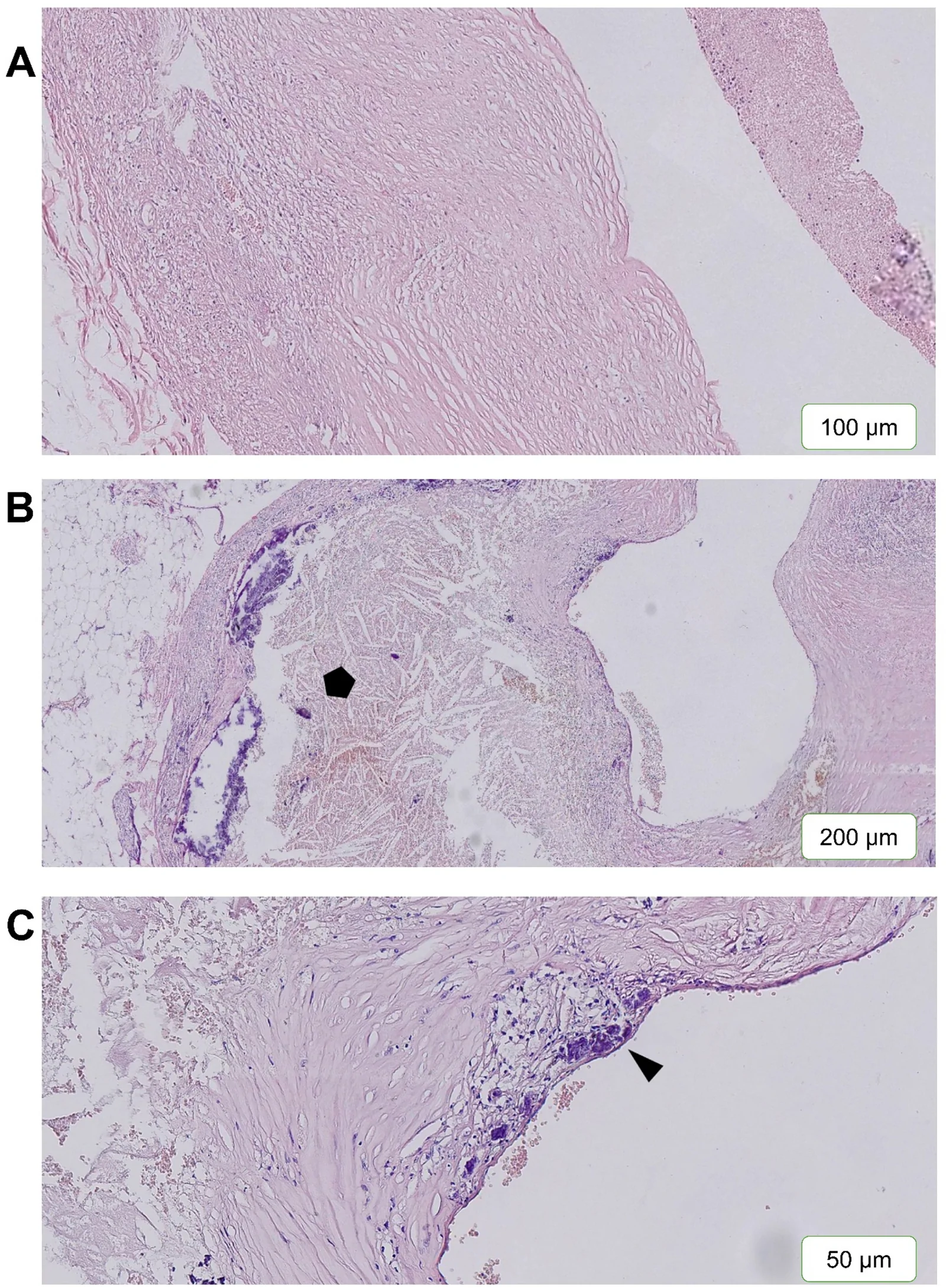
Representative histopathological features of coronary arteries in control and hypertensive cohorts. **A** Coronary artery wall from a patient in the control group. The arterial wall shows no histopathological abnormalities. **B **Coronary artery from a patient in the hypertension group, demonstrating a fibrocalcifiс atherosclerotic plaque with a central necrotic core and peripheral calcifications (pentagon). The atherosclerotic plaque causes marked narrowing of the arterial lumen. **C** Atherosclerotic plaque, high magnification. A subintimal calcification with macrophage infiltration (arrowhead) is present; scattered macrophages with pale cytoplasm (foam cells), are also observed. Hematoxylin and eosin (H&E) stain. Scale bars: 100 μm (**A**), 200 μm (**B**), and 50 μm (**C**)

To further investigate the pathogenetic basis of vascular remodeling at the tissue level, our quantitative analysis was supplemented with a qualitative visual assessment of the microsections. First, general vessel morphology and atherosclerotic changes were assessed using H&E staining. This evaluation demonstrated intact arterial walls in the control group, contrasting with pronounced structural disruption, including fibrocalcific plaques and macrophage infiltration, in the hypertensive cohort (Fig. 2).

**Fig. 3:**
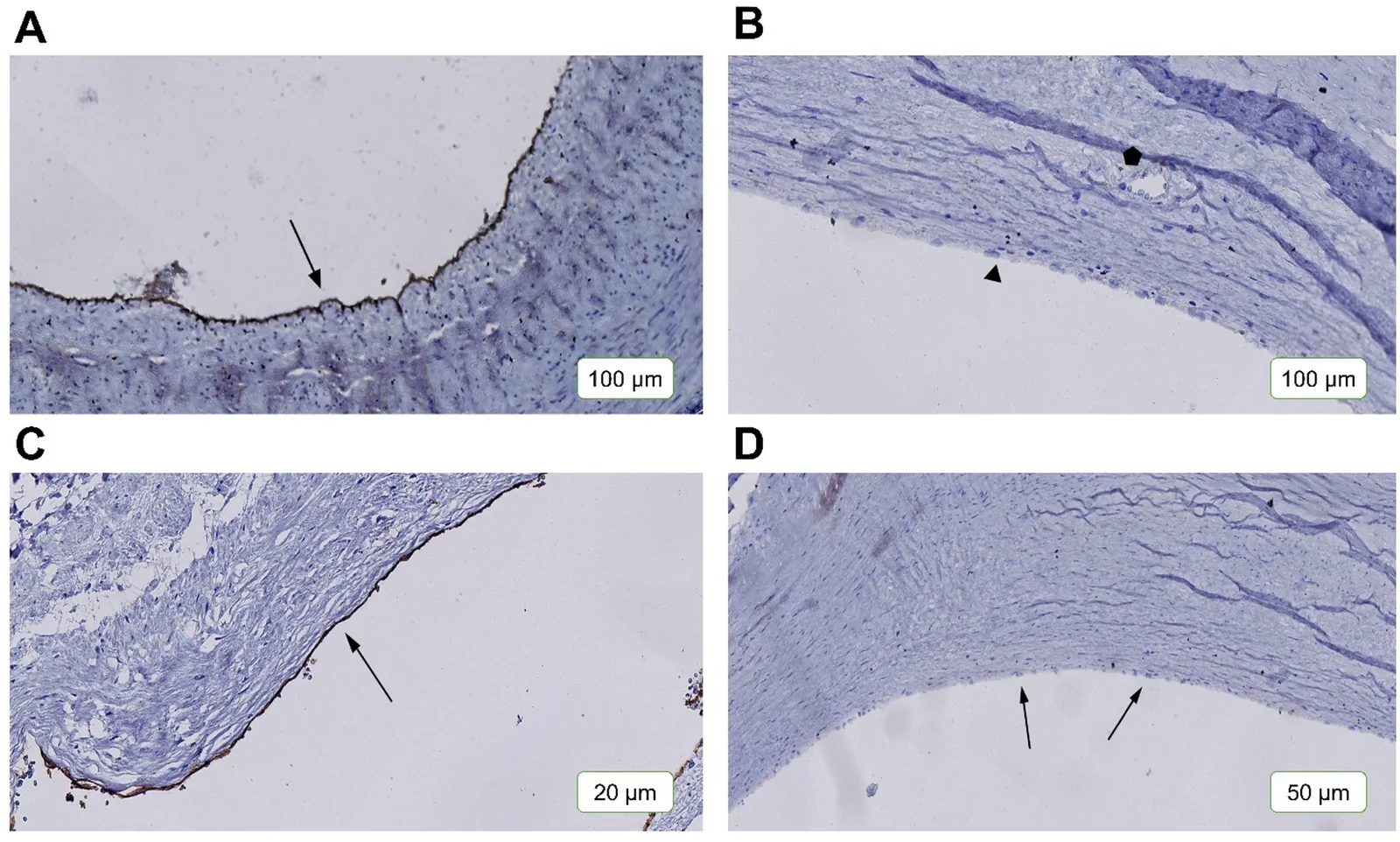
Immunohistochemical analysis of endothelial CD31 expression in coronary arteries. **A** Fragment of the coronary artery wall from a patient in the control group. CD31 deposits (brown staining) are clearly visualized in the endothelial layer (arrow). **B** Coronary artery wall from a patient in the hypertension group. The endothelium shows no CD31 deposits (arrowhead), nor does the small capillary within the vessel wall (pentagon). **C** Patient from the hypertension group; deposits of CD31 (brown staining) are clearly visualized in the endothelial layer (arrow). **D** Coronary artery wall from a patient in the control group. The endothelium shows no CD31 deposits (arrows). Immunohistochemical staining for CD31 with hematoxylin counterstain. Scale bars: 100 μm (**A**, **B**), 20 μm (**C**), and 50 μm (**D**)

Subsequent immunohistochemical evaluation focused on the physical integrity of the endothelial monolayer. The specific spatial distribution patterns of CD31 confirmed varying degrees of endothelial preservation and focal cellular loss across the study cohorts (Fig. 3).

**Fig. 4:**
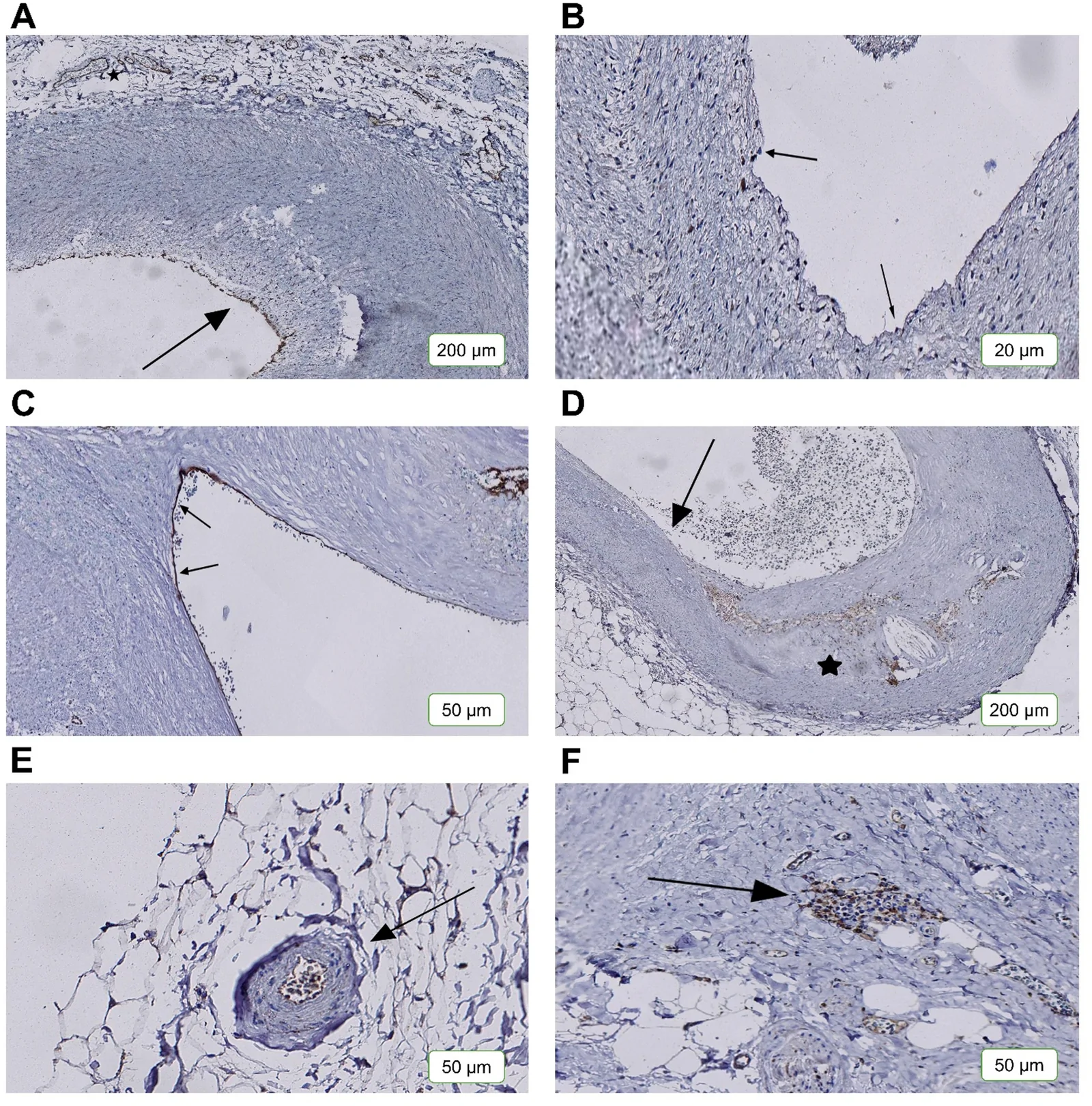
Immunohistochemical evaluation of CD138 expression in the coronary artery wall and adjacent tissues. **A** A fragment of the coronary artery wall from a control group patient; CD138 deposits (brown color) are clearly visualized in the endothelium (arrow) and in the capillaries (star) of the adjacent adipose tissue. **B** Coronary artery wall from a patient in the control group, demonstrating an area where the endothelium shows no CD138 deposits (arrows). **C** Patient from the hypertension group; deposits of CD138 (brown staining) are clearly visualized in the endothelial layer (arrows). **D** Patient from the hypertension group showing a coronary artery with an atherosclerotic plaque; within the atherosclerotic plaque, lipid deposits, cholesterol crystals, and infiltration by foamy macrophages (star) are visualized, while no CD138 deposits are detected in the endothelium (arrow). **E** Patient from the hypertension group; an arteriole (arrow) in the adjacent adipose tissue shows marked wall thickening and features of arteriolosclerosis, with CD138 deposits visualized in the endothelium. **F** Patient from the hypertension group, illustrating infiltration by CD138-positive (brown color) plasma cells (arrow) within the wall of the coronary artery. Immunohistochemical staining for CD138 with hematoxylin counterstain. Scale bars: 200 μm (**A**, **D**), 20 μm (**B**), and 50 μm (**C**, **E**, **F**)

Finally, to assess the status of the endothelial glycocalyx and adjacent tissues, we evaluated the expression of CD138. This analysis illustrated the complex morphological spectrum of syndecan-1 localization, ranging from preserved perivascular capillaries to complete focal depletion over advanced atherosclerotic plaques (Fig. 4).

**Fig. 5:**
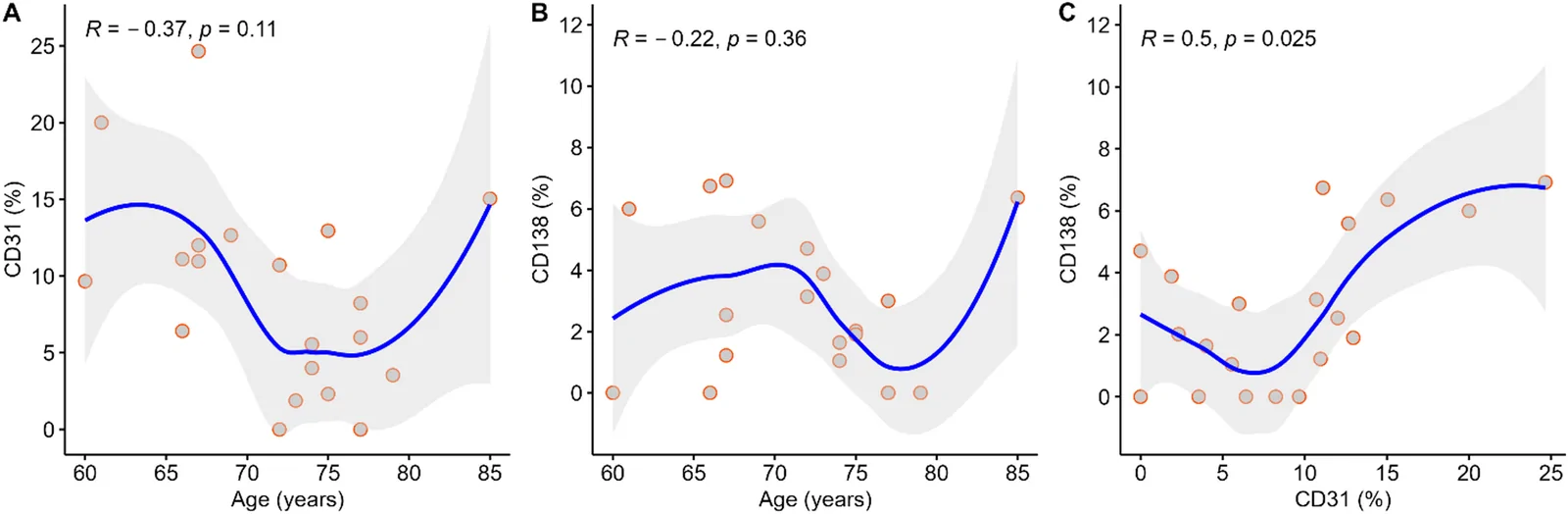
Spearman correlation analysis of age and morphological markers in the hypertensive cohort. Scatter plots illustrate the bivariate relationships between **A**, age and CD31 expression area; **B**, age and CD138 expression area; and **C**, CD31 and CD138 expression areas. Data points represent individual patients within the primary study group (*n* = 20). The blue curves represent Locally Estimated Scatterplot Smoothing (LOESS) trend lines to visualize underlying non-linear trajectories, surrounded by gray shading indicating 95% confidence intervals. Spearman’s rank correlation coefficients (R) and corresponding two-sided P values are embedded within each panel.

While the comprehensive correlation matrix encompassing all clinical and laboratory parameters is accessible via the project’s public repository (see Data Availability statement), our primary analysis focused on the key relationships between patient age and the expression of vascular markers within the hypertensive cohort (Fig. 5).

Bivariate Spearman’s rank-order analysis revealed a weak-to-moderate inverse trend between age and the expression areas of both markers; however, these isolated monotonic associations did not reach statistical significance (CD31: *R* = -0.37, *p* = 0.11; CD138: *R* = -0.22, *p* = 0.36). Conversely, a robust and statistically significant positive correlation was identified between the expression levels of the endothelial marker CD31 and the glycocalyx marker CD138 (*R* = 0.50, *p* = 0.025). This synchronized expression pattern highlights a statistical association between the structural degradation of the endothelium and its overlying glycocalyx, though further mechanistic studies are needed to confirm a direct biological coupling during vascular remodeling.

**Fig. 6:**
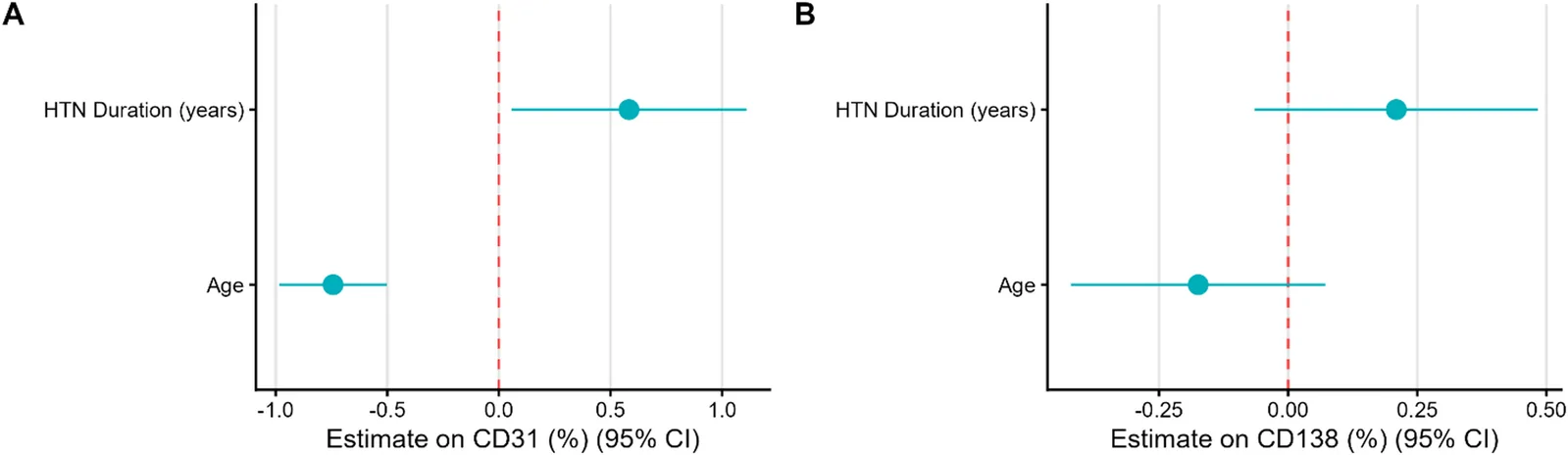
Forest plots of multivariable linear regression models evaluating predictors of endothelial and glycocalyx markers. The plots display the unstandardized regression coefficients and their corresponding 95% confidence intervals for **A**, the CD31 expression model, and **B**, the CD138 expression model. The red dashed line represents the null effect (Beta = 0). Predictors whose confidence intervals do not cross the zero line are considered statistically significant. For the CD31 model (Panel **A**), confidence intervals and significance were adjusted using HAC robust standard errors to account for residual autocorrelation.

To evaluate the independent effects of clinical and demographic factors on endothelial dysfunction and glycocalyx degradation, multiple linear regression models were constructed within the hypertensive cohort (Table 2; Fig. 6). Patient age and the duration of hypertension were included as independent variables. The absence of multicollinearity was confirmed using the variance inflation factor (VIF < 2.0).

**Table 2:** Multivariable linear regression models evaluating factors associated with the expression areas of morphological markers in the hypertensive cohort (*n* = 20)

Predictor	CD31 Model (Expression Area, %)		CD138 Model (Expression Area, %)	
	Beta (95% CI)	*P* value	Beta (95% CI)	*P* value
(Intercept)	53.00 (38.00 to 67.00)	< 0.001	12.00 (-3.30 to 27.00)	0.120
Age (years)	-0.74 (-0.98 to -0.50)	0.016	-0.17 (-0.42 to 0.07)	0.200
HTN Duration (years)	0.58 (-0.06 to 1.10)	0.076	0.21 (-0.06 to 0.48)	0.130
**Model Diagnostics**				
R² / Adjusted R²	0.298 / 0.215		0.147 / 0.047	
Overall Model P value	0.049		0.300	

Beta represents the unstandardized regression coefficient. CI indicates the 95% confidence interval. Significant P values (< 0.05) are highlighted in bold. *HTN* Hypertension. Due to identified residual autocorrelation (Durbin-Watson *p* = 0.0077), the 95% CIs and *P* values for the CD31 model were calculated using heteroskedasticity and autocorrelation consistent (HAC) robust standard errors. The CD138 model met all assumptions for ordinary least squares regression

The predictive model for CD31 expression was statistically significant (*p* = 0.049), accounting for 21.5% of the variance in the marker’s expression area (Adjusted R² = 0.215). Due to the detection of residual autocorrelation during model diagnostics (Durbin-Watson *p* = 0.0077), Newey-West heteroskedasticity and autocorrelation consistent (HAC) robust standard errors were applied to ensure reliable inference. The analysis identified age as an independent and significant associated factor: each one-year increase in age was associated with a 0.74% decrease in CD31 expression area (95% CI: -0.98 to -0.50, *p* = 0.016). The duration of hypertension did not demonstrate a statistically significant independent association with CD31 expression area (β = 0.58, 95% CI: -0.06 to 1.10, *p* = 0.076).

Conversely, the regression model for CD138 expression did not achieve overall statistical significance (*p* = 0.30, Adjusted R² = 0.047), despite fully meeting all linear regression assumptions (Shapiro-Wilk *p* = 0.36; Breusch-Pagan *p* = 0.56). Neither age (*p* = 0.20) nor hypertension duration (*p* = 0.13) demonstrated a significant independent effect on CD138 expression in this multivariable model.

## Discussion

The primary objective of this study was to evaluate the spatial expression of endothelial (CD31) and glycocalyx (CD138) markers in coronary arteries and to disentangle the effects of chronological age and hypertension duration. Our central finding is that chronological age, rather than the chronicity of hypertension, emerges as a significant independent factor associated with reduced CD31 expression within the hypertensive cohort. The multivariable regression analysis demonstrated a significant inverse relationship between age and CD31 expression area. This aligns closely with recent global literature emphasizing the concept of “endothelial senescence,” whereby physiological aging inherently drives the progressive loss of endothelial tight junctions and adhesion molecules [16–19]. Several robust cohorts have recently shown that aging microvasculature exhibits impaired angiogenesis and a natural decline in CD31 density, independent of systemic blood pressure [20, 21]. Our post-mortem tissue data corroborate these clinical observations, suggesting that the integrity of the CD31-positive endothelial monolayer is strongly age-dependent. However, we acknowledge that defining the comprehensive structural vulnerability of the coronary artery based solely on CD31 expression is limited. Further evaluation incorporating additional morphometric parameters of structural remodeling (such as intimal thickness and media-to-lumen ratio) is necessary to fully confirm the extent of pressure-induced vascular damage.

Secondly, while the multivariable model for CD138 did not reach statistical significance, we observed a robust positive correlation between the tissue expression levels of CD31 and CD138. This synchronized expression pattern supports a spatial association between endothelial disruption and glycocalyx shedding [22, 23]. However, since neither chronological age nor hypertension duration was independently associated with CD138 loss in our multivariable analysis, defining this interaction strictly as a primary structural mechanism would be premature. Further studies incorporating direct quantitative histopathological parameters of vascular remodeling (such as intimal thickening or matrix fibrosis) are required to establish the precise mechanistic pathways of this structural degradation. Current literature predominantly focuses on the acute shedding of the glycocalyx into the bloodstream, measuring soluble syndecan-1 during acute hypertensive crises or sepsis [24–26]. In contrast, our in situ tissue analysis reflects the chronic state of the arterial wall. The lack of an independent association between hypertension duration and CD138 expression, combined with the absence of significant baseline differences compared to the control cohort, suggests that tissue-level glycocalyx integrity is highly variable. Rather than following a linear temporal decline or reflecting early rapid depletion, localized CD138 degradation may be more dependent on superimposed acute cardiometabolic or inflammatory stressors rather than the sheer chronicity of hemodynamic overload [27, 28]. This highlights a crucial discrepancy between dynamic circulating biomarkers and static tissue morphology, necessitating a cautious interpretation of purely serological studies.

Crucially, our multivariable regression model demonstrated that the duration of hypertension was not a statistically significant independent associated factor for CD31 expression area (*p* = 0.076). While previous literature indicates that prolonged hypertension can induce adaptive structural remodeling, such as compensatory neoangiogenesis and microvascular hyperplasia in an attempt to restore impaired perfusion [29–33], our statistical outcome strongly reinforces our primary hypothesis: within this specific patient cohort, chronological age—rather than the prolonged chronicity of the disease itself—acts as the primary independent driver of coronary endothelial degradation. This phenomenon underscores the complexity of interpreting cross-sectional morphological data, where severe age-related endothelial rarefaction may be partially masked by pathological, dysfunctional hyperproliferation driven by chronic hemodynamic overload [3, 34]. The initial lack of significance in direct intergroup comparisons underscores the necessity of our multivariable approach to control for age-related confounding bias.

It is important to emphasize that these luminal alterations do not occur in isolation. Vascular remodeling in aging and essential hypertension is a deeply multifactorial process that also involves profound extracellular matrix reorganization and progressive arterial stiffening [35]. The structural degradation of the CD31-positive endothelial monolayer and the shedding of the CD138-rich glycocalyx are likely closely intertwined with deeper intimal and medial changes, such as pathological collagen deposition, elastin fragmentation, and the accumulation of advanced glycation end-products [36]. Furthermore, remodeling of the basement membrane and alterations in other structural proteoglycans, such as perlecan, play critical roles in mechanotransduction and maintaining the integrity of the vascular wall [37]. The crosstalk between the superficial glycocalyx disruption and the underlying profound extracellular matrix stiffening creates a pathological feedback loop that exacerbates microvascular dysfunction and accelerates both age-induced and hypertension-mediated vascular injury [38].

Several limitations of this study should be acknowledged.

First, the investigation is constrained by a relatively small sample size (*n* = 30), which is inherent to post-mortem histopathological studies utilizing archived autopsy material. Specifically, working with autopsy specimens introduces the unavoidable risk of focal post-mortem endothelial desquamation due to early autolysis or mechanical disruption during harvesting, which may lead to extreme minimal values of marker expression in isolated cases regardless of baseline physiological status. Furthermore, the small sample size restricted the number of variables that could be safely included in our multivariable regression models without risking severe overfitting. Consequently, we could not adjust for several important clinical covariates, such as body mass index, smoking history, diabetes mellitus, dyslipidemia, and chronic antihypertensive medication use, which are known independent modulators of endothelial and glycocalyx integrity. Due to the retrospective nature of the terminal inpatient records, retrieving continuous and reliable ambulatory data for these cardiometabolic confounders was not feasible. Therefore, the observed morphological changes must be interpreted with the caveat that these unmeasured factors may exert residual confounding effects. Consequently, the design is primarily exploratory. While the use of robust statistical methods and 95% confidence intervals confirmed the reliability of the observed significant effects regarding age, the lack of statistical significance regarding the duration of hypertension may reflect limited statistical power rather than a true absence of biological effect. Therefore, these preliminary findings warrant further validation in larger, multicenter cohorts.

Second, the substantial age discrepancy between the control and hypertensive groups precluded simple direct comparisons. The absence of an age-matched normotensive control group represents an important limitation. However, this study design was dictated by the severe constraints of post-mortem material collection: finding elderly autopsy subjects with a confirmed absence of arterial hypertension and cardiovascular disease is practically impossible. Therefore, the use of a younger, conditionally healthy control group was a necessary compromise to establish a baseline of intact vascular morphology. While we rigorously addressed this confounding bias mathematically by restricting the multivariable regression analysis exclusively to the hypertensive cohort, the lack of an ideal age-matched baseline limits our ability to fully definitively disentangle the earliest stages of hypertension-mediated remodeling from physiological aging.

Third, our morphological assessment relied on the quantitative evaluation of single 2D histological cross-sections. While utilizing whole-slide imaging allowed us to analyze the entire continuous endothelial perimeter of each cross-section rather than isolated microscopic fields, 2D sections inherently cannot capture the full 3D spatial heterogeneity of vascular remodeling and focal glycocalyx shedding along the longitudinal axis of the artery.

Fourth, our quantitative assessment was restricted exclusively to the left coronary artery system. Given the distinct biomechanical stress and variable hemodynamic conditions experienced by the right coronary artery, the degree of endothelial degradation and structural remodeling may differ significantly between these regional localizations. Evaluating these inter-arterial differences represents a crucial vector for future morphological studies. Furthermore, our manual annotation protocol combined the endothelial and subendothelial matrix into a single region of interest to calculate relative marker expression, precluding the isolated morphometric analysis of intimal thickening. Future studies employing 3D volumetric tissue reconstruction or advanced light-sheet microscopy are necessary to map these topographical dynamics comprehensively.

## Conclusions

In conclusion, this in situ immunohistochemical analysis indicates that chronological age is a significant independent factor associated with reduced CD31 expression in the coronary arteries of hypertensive patients, reflecting age-related structural endothelial alterations. Furthermore, the significant positive correlation between CD31 and CD138 expression suggests a synchronized spatial pattern of degradation, indicating that the structural integrity of the endothelium and its protective glycocalyx are closely associated during hypertensive vascular injury. Our findings emphasize that future investigations of vascular remodeling must critically separate the inherent effects of physiological senescence from the pathological impact of chronic hemodynamic overload. Further large-scale studies combining in situ morphological assessment with circulating biomarkers are required to fully elucidate the timeline of glycocalyx shedding and endothelial decay in essential hypertension.

## Supplementary Information

Supplementary Material 1.

## Data Availability

The datasets generated and analyzed during the current study, along with the complete R scripts for statistical modeling and visualization, are publicly available in the GitHub repository (https://github.com/NefroStat/morphological-htn-study) and archived in Zenodo with the https://doi.org/10.5281/zenodo.19626466.

## Abbreviations

ALT: Alanine Aminotransferase
AST: Aspartate Aminotransferase
CD31: Platelet Endothelial Cell Adhesion Molecule-1 (PECAM-1)
CD138: Syndecan-1
CI: Confidence Interval
HAC: Heteroskedasticity and Autocorrelation Consistent
H&E: Hematoxylin and Eosin
HTN: Hypertension
IHC: Immunohistochemistry
IQR: Interquartile Range
DAB: 3,3’-Diaminobenzidine
RGB: Red, Green, Blue
