# Supplementary material for: Age, rather than hypertension duration, drives coronary endothelial degradation: an in situ post-mortem analysis
Source: BMC Cardiovasc Disord. 2026 Jul 1;26:813. doi: 10.1186/s12872-026-06210-z (PMC13587489; doi:10.1186/s12872-026-06210-z)
Supplement: Supplementary file 1 [file 12872_2026_6210_MOESM1_ESM.docx]

**Supplementary Figure S1. Optimization and validation of the optical density threshold for digital image analysis in QuPath.**

(A) Original representative brightfield image of the coronary artery wall stained with DAB chromogen, demonstrating specific endothelial expression and background tissue counterstained with hematoxylin. (B) QuPath segmentation mask applied with a low OD threshold of 0.15. The red overlay indicates positive pixel detection, revealing significant false-positive background noise and non-specific chromogen binding in the subendothelial stroma. (C) QuPath segmentation mask applied with the optimized strict OD threshold of 0.35. The red overlay demonstrates highly specific and accurate detection of true positive DAB signals restricted strictly to the endothelial layer, completely eliminating non-specific background noise.
